# Online Centralized MPC for Lane Merging in Vehicle Platoons

**DOI:** 10.3390/s25175605

**Published:** 2025-09-08

**Authors:** Shila Alizadehghobadi, Mukesh Singhal, Reza Ehsani

**Affiliations:** 1 Department of Mechanical Engineering, University of California, Merced, CA 95343, USA; 2 Department of Electrical Engineering and Computer Science, University of California, Merced, CA 95343, USA

**Keywords:** online, platoon, lane merging, centralized MPC, prediction horizon, reordering

## Abstract

In the context of autonomous vehicles, proper lane merging is critical as it can reduce the traffic bottleneck and lead to safer road transportation. To obtain a collision-free and efficient lane merging, advanced control algorithms need to be designed to smoothly coordinate multiple vehicles to form a platoon. Model predictive control (MPC) is such a controller capable of forecasting future states of multiple vehicles by optimizing their control inputs while satisfying the constraints. Prior MPC-based studies mostly utilized offline planning with a precomputed lookup table of feasible maneuvers to model lane merging. Although these model designs reduce the online computational load, they lack flexibility, as they rely on predefined scenarios and cannot easily adapt to dynamic or unpredictable situations. In this study, we present a centralized MPC framework capable of online trajectory tracking under dynamic constraints and disturbances, for collision-free operation in tightly spaced multi-vehicle platoons. To evaluate the flexibility of our online algorithm, we examine the role of prediction horizon—the time window over which future states are forecasted—and platoon size in determining both the feasibility and efficiency of merging maneuvers. Our results reveal that there exists an optimal prediction horizon at which braking and acceleration can be minimized, thereby reducing energy consumption by 35–40%. Additionally, we observe that increasing the prediction horizon beyond the minimum required for feasibility can alter the vehicle sequence in the platoon. Capturing the changes in vehicle sequence (e.g., who leads or yields) when prediction horizon varies, is a consequence of online trajectory optimization. This vehicle sequence change cannot be captured by offline planning that relies on precomputed look-up table maneuvers. We also found that as the number of vehicles increases, the minimum feasible prediction horizon increases significantly.

## 1. Introduction

With the growing interest in autonomous vehicles, the number of these vehicles on the roads is increasing, leading to significant traffic congestion, higher energy consumption, and a higher risk of accidents [1,2,3]. Some of the common traffic situations are road closures, highway exits, and on-ramp merging [4,5], where lane merging and platoon formation are inevitable. In these situations, a proper lane merging and efficient platooning are critical to avoid the risk of accidents, and reduce traffic congestion and fuel consumption [6,7]. Smooth and safe coordination of multiple vehicles to form a platoon necessitates designing advanced controllers—that is, algorithms or systems that compute and apply control inputs (e.g., acceleration, braking, steering) to ensure desired vehicle behavior under certain constraints. The simplest case of platooning is when a number of vehicles form a train in a single lane [8]. Design and experimental implementation of a controller for single-lane platooning were demonstrated in a few studies [9,10]. Later, studies focused on designing controllers to maintain a desired acceleration and safe string-stable operation (i.e., a condition in which disturbances such as sudden acceleration or braking smoothly propagate along the vehicle platoon) even when vehicles travel very short inter-vehicle distances [11,12,13]. These controller designs shed light on the development of vehicle platooning models for more complex strategies, such as lane merging in multi-lane roads [14,15].

Owing to the fact that in the context of multiple vehicle platoons with small inter-vehicle distances, collision avoidance is the most critical factor, optimization-based methods that can address collision safety in platooning have lately received a great deal of attention [16,17]. Recently, optimization-based controllers based on MPC have been used to guarantee collision avoidance by optimizing the trajectories where the collision avoidance is set as a constraint of the trajectory optimization problem [18,19,20,21,22].

One of the major factors in optimally designing an MPC to avoid collision in a platoon formation is the prediction horizon. The prediction horizon is the time duration over which the future states of the system are predicted. Choosing a longer prediction horizon reduces the risk of collision; however, it increases the controller’s computational load [23,24,25,26]. Li et al. [27] employed an MPC with a variable prediction horizon that adapts to changing driving conditions for an individual vehicle (i.e., not a platoon). Their results showed that using a variable prediction horizon improved prediction accuracy by 7%. Chen et al. [26] considered a varying prediction horizon in an MPC for a single vehicle and observed that it delivers a superior tracking performance and robustness compared to a fixed prediction horizon. Other studies were also conducted to investigate the effect of variable horizons on the MPC [28,29].

While ensuring safety through appropriate prediction horizons is critical, it is only one aspect of MPC optimization for platooning and lane merging. Once collision avoidance is reliably achieved, attention can shift toward improving the energy efficiency of the control system. This has led to the development of new control strategies for lane merging that consider energy efficiency [30,31]. In a study, Nie and Farzaneh [30] introduced a lane-changing motion planning approach aimed at achieving personalized and energy-efficient autonomous driving. Their method formulated the planning task as a constrained optimization problem, using quintic polynomials to represent a family of smooth and flexible trajectories. A quintic polynomial, which is a mathematical expression capable of capturing continuous changes in position, velocity, and acceleration, was used to ensure comfort and physical feasibility during lane changes. By treating the lane-changing duration as a key decision variable, their approach selected the trajectory that minimized energy consumption while aligning with individual driving styles.

Yue et al. [30] propose a centralized sequencing game combined with distributed vehicle control for ecological platoon merging. Their approach prescribes a fixed merging sequence which is determined in advance and remains unchanged as the vehicles follow their designated trajectories. However, in real-world scenarios, the merging sequence is often dynamic and depends on the interactions and cooperation between vehicles.

In intelligent connected traffic systems, infrastructure (e.g., traffic lights and sensors) and vehicles communicate online using precise communication technologies. This allows coordination between higher-level systems (e.g., traffic control centers or cloud-based platforms) and lower-level vehicle systems (e.g., the vehicle’s engine, brakes, and controllers). By integrating these systems, vehicles can operate more efficiently, for example, by adjusting the route based on traffic flow, which helps reduce energy consumption [31,32]. Turri et al. [33] utilized a dynamic programming approach to obtain the optimal energy as a function of velocity of the platoon using a distributed MPC. They claimed that in comparison to the standard platoon controllers, their controller design could save up to 12% energy for the following vehicles.

In an attempt to enhance energy efficiency of hybrid electric vehicles, Yu et al. [34] employed an MPC based on the information of drag forces and road gradients. Their simulation suggested that energy could be saved due to both air drag reduction and an appropriate controller. Ma et al. [35] proposed a predictive optimization strategy based on a nonlinear MPC. Their strategy passed different standards of energy saving tests including the Urban Driving Cycle (UDC), highway fuel economy test (HWFET), and new European driving cycle (NEDC), saving energy by 16.1%, 6.2%, and 11.7%, respectively. Zhan et al. [36] proposed an MPC with the objective function to minimize platoon duration and consequently energy consumption. Their results showed that the overall energy consumption decreased by 21.3%. For energy-saving purposes, Ma et al. [37] designed a multi-layer energy-efficient cooperative adaptive cruise control (ECACC) scheme that integrated dynamic programming and feedforward/feedback control for platooning. Their controller could reduce energy consumption compared to the traditional energy-optimized cruise systems. He et al. [38] developed a signal eco-approach (SEA) that optimized the velocity of platoon vehicles at multi-signal intersections. Han et al. [39] proposed a trajectory optimization approach aimed at minimizing the overall fuel consumption of a fleet of connected and automated vehicles (CAVs) at signalized intersections. By applying predictive trajectory optimization, they derived optimal paths for vehicles in queues, demonstrating notable reductions in both travel time and fuel usage.

Here, we introduce an online (i.e., on-the-fly) centralized MPC for energy-efficient multi-vehicle platooning, capturing the vehicle reordering as a result of changing the prediction horizon under dynamic constraints and disturbances. This vehicle reordering could regulate the energy used by the vehicles through braking/acceleration. Additionally, unlike previous works [14] where vehicle orientation (i.e., heading angle) is handled indirectly through precomputed geometry, our method incorporates online, orientation-aware collision avoidance directly into the optimization formulation. This enables the collision avoidance constraints to adapt dynamically at each time step based on the current orientation of every vehicle. This is particularly critical for handling complex interactions during lane merging, where online orientation changes can determine whether safe and feasible trajectories exist. Our approach allows the solver to exploit these orientation-dependent dynamics to naturally produce emergent behaviors like vehicle reordering.

The rest of the paper is structured as follows. Section 2 presents the methodology including the mathematical model of the system (Section 2.1), motion planning (Section 2.2), and energy saving (Section 2.3). Section 3 presents and discusses the simulation results that are performed for platoons with two, four, and six vehicles. Section 4 covers the sensitivity analysis. Section 5 discusses the limitations and future direction, and Section 6 concludes the paper with final remarks.

## 2. Methodology

This study presents an online centralized MPC framework for multi-vehicle platooning that ensures collision avoidance and optimal trajectory tracking under dynamic constraints and disturbances. The term online refers to the capability of the centralized MPC controller to solve the constrained optimization problem at every time step using updated vehicle states and current conditions, without relying on precomputed trajectories. Using the online approach is crucial as it enables adaptation to online changes within the platoon environment, such as prediction horizon variation and reordering of vehicle sequences. By incorporating such flexibility, the controller can adjust the prediction horizon and vehicle sequence based on each vehicle’s conditions. In offline motion planning, the trajectory is decided without a prediction horizon and assuming the velocity and the inter-vehicle distance are fixed. The methodology is outlined below:

- First, we generate the reference trajectory offline by giving the initial and the final configuration of the vehicles. This trajectory generation uses a linear function for transition of vehicles from their initial (upper) to final (lower) lane positions. This generation of a reference trajectory is computed outside of the MPC and does not take into account collisions between vehicles.
- The reference trajectory is then used in the online centralized controller (i.e., MPC) which solves an optimization problem with respect to state, input, and collision avoidance constraints. The MPC recalculates the optimal inputs at every time step using updated measurements, rather than relying on a pre-planned trajectory that may no longer be optimal. This allows minimal acceleration and braking. The controller finally delivers the computed optimal acceleration, braking, and steering to the vehicles.

### 2.1. Dynamic Model of the Vehicle

**Figure 1:**
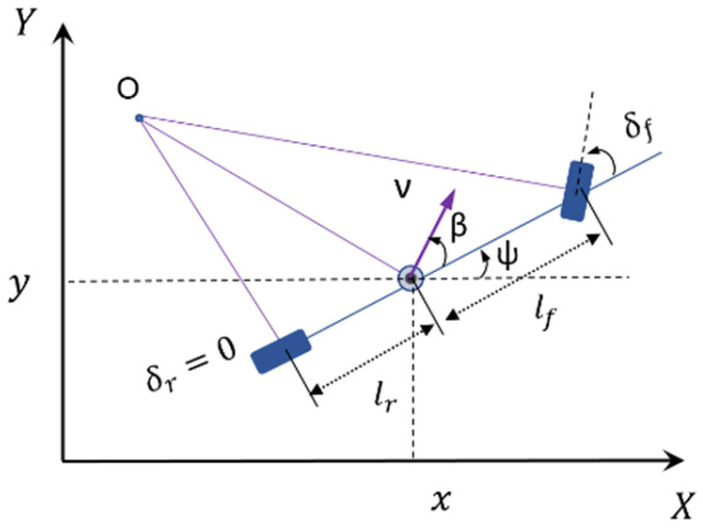
Kinematic bicycle model.

The MPC is a model-based controller where a mathematical model is incorporated into the controller. Vehicles are typically modeled with a dynamic bicycle model or kinematic bicycle model [40,41]. The four-wheel dynamic model completely captures the behavior of the vehicles; however, it is associated with complexities in terms of nonlinearity and the number of equations [40]. This model is essential for simulating vehicles operating at extreme limits, such as in drifting or racing scenarios, where they navigate sharp turns and reach high speeds. On the other hand, the kinematic bicycle model, as shown in Figure 1, is a simplified version of the dynamic bicycle model, conserving significant computational resources by disregarding forces and moments. This model is well-suited for urban driving speeds and maneuvers, where limitations on the steering angle and vehicle speed make it adequate for typical city driving, unlike the demands of racing vehicles.

In our method, the dynamics of a vehicle are described using the kinematic bicycle model, which transforms the complex interaction of forces and motions into a simplified form. This model considers the vehicle as a two-wheeled system (see Figure 1), capturing the essential dynamics for control and optimization purposes. The model assumes that the steering angle ($\delta_{f}$) is relatively small and there is no significant wheel slip. The mathematical model of a vehicle described by the kinematic bicycle model can be written as follows [41]:

(1) $${\dot{x}}^{i}=v^{i}cos\left(\psi^{i}+\beta^{i}\right)$$

(2) $${\dot{y}}^{i}=v^{i}sin\left(\psi^{i}+\beta^{i}\right)$$

(3) $${\dot{\psi }}^{i}=\frac{v^{i}cos\beta^{i}}{l_{f}+l_{r}}tan\delta^{i}$$

(4) $${\dot{v}}^{i}=a^{i}$$

$x^{i}$, $y^{i}$, $\psi^{i}$, $v^{i}$, and $a^{i}$ stand for longitudinal position, lateral position, heading angle, and velocity and acceleration at the center of gravity (C.G.) of the i-th vehicle in the platoon. $\beta^{i}=arctan\left(\frac{\tan\delta^{i}l_{r}}{l_{f}+l_{r}}\right)$ refers to the side slip angle $l_{f}$ and $l_{r}$ are the distance from C.G. to the front and rear axles, respectively. The equations of the kinematic bicycle model can be rewritten in a discrete-time format by using a forward Euler discretization as [14]:

(5) $$x^{i}\left(t+1\right)=x^{i}\left(t\right)+\Delta t\;v^{i}\left(t\right)cos\left(\psi^{i}(t)+\beta^{i}(t)\right)$$

(6) $$y^{i}\left(t+1\right)=y^{i}\left(t\right)+\Delta t\;v^{i}\left(t\right)sin\left(\psi^{i}(t)+\beta^{i}(t)\right)$$

(7) $$\psi^{i}\left(t+1\right)=\psi^{i}\left(t\right)+\Delta t\frac{v^{i}sin\left(\psi^{i}+\beta^{i}\right)}{l_{if}+l_{ir}}tan\delta^{i}\left(t\right)$$

(8) $$v^{i}\left(t+1\right)=v^{i}\left(t\right)+\Delta t\;a^{i}\left(t\right)$$

with Δt being the time interval of the Euler iterations in seconds.

### 2.2. Motion Planning of Vehicles

**Figure 2:**
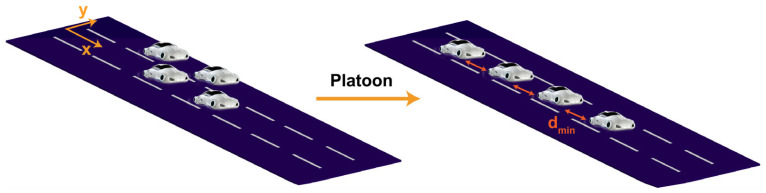
Schematic of the vehicles before (initial configuration) and after (final configuration) forming the platoon.

The problem configuration consists of the vehicles in two lanes (as the initial configuration) that are merging into a single lane (final configuration), forming a platoon (Figure 2). The motion planning comprises an offline motion planner and an online control system. Based on the initial and final configurations of the platoon, the offline motion planner generates reference trajectories that connect the initial configuration to the final configuration.

**Figure 3:**
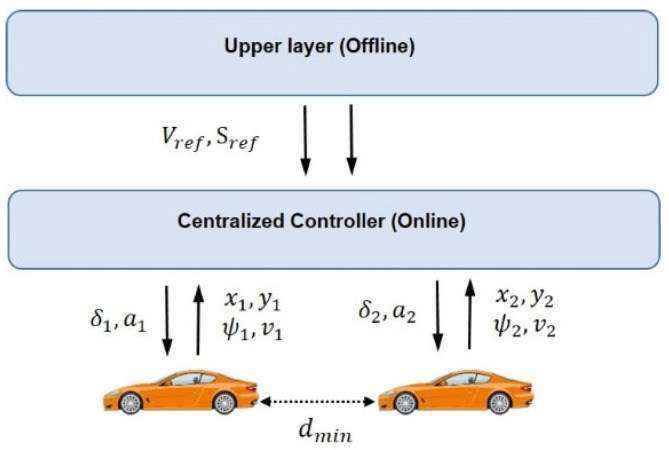
Schematic for the centralized controller with two vehicles.

The online controller then handles online adjustment to the reference trajectories based on the precomputed trajectories from the offline motion planner and the collision avoidance constraints (Figure 3). This online adjustment is performed by generating the control inputs (i.e., acceleration and steering angle) to guide the vehicles along their designated trajectories. This stage translates upper-level planning (offline motion planner) into lower-level (online) control commands that adjust vehicle states (Equations (5)–(8)) through constraint finite-time optimal control (CFTOC) optimization (explained later). Finally, the control inputs are sent to the vehicles, ensuring they follow the optimized trajectories generated by the offline planning and adjusted by the online control system.

Generating appropriate reference trajectories is critical for effective lane merging. We use an integrator-based approach to generate the reference trajectories that accommodate various platoon sizes. The offline motion planning step generates a reference trajectory that guides each vehicle in the platoon toward its desired configuration. This reference trajectory is calculated outside the MPC. It is important to note that the offline trajectory does not account for collision avoidance. Instead, the MPC handles dynamic constraints, including inter-vehicle safety distance, during online control. The reference trajectory is generated for the time to form the platoon, and can be easily extended to accommodate different platoon sizes. The reference trajectories for various platoon sizes and configurations were generated and then are used in an online centralized MPC for multi-vehicle motion planning and calculating the inputs and states at each time step using CFTOC:

(9) $$\underset{u_{t}^{i}\left(\left.\cdot \right|t\right),\lambda_{\mathrm{ij}}\left(\left.\cdot \right|t\right),s_{\mathrm{ij}}\left(\left.\cdot \right|t\right)}{\min}\sum_{i=1}^{N_{V}}\left(\sum_{k=t}^{t+N}{\left\|Q_{z}\left(z^{i}\left(\left.k\right|t\right)-z_{Ref}^{i}\left(\left.k\right|t\right)\right)\right\|}_{2}^{2}+\;\sum_{k=t}^{t+N-1}{\left\|Q_{u}\left(u^{i}\left(\left.k\right|t\right)\right)\right\|}_{2}^{2}+{\left\|Q_{\Delta u}\left(\Delta u^{i}\left(\left.k\right|t\right)\right)\right\|}_{2}^{2}\right)$$

(9a) $$z^{i}\left(\left.k+1\right|t\right)=f\left(z^{i}\left(\left.k\right|t\right),u^{i}\left(\left.k\right|t\right)\right)$$

(9b) $$z^{i}\left(\left.0\right|t\right)=z^{i}\left(t\right)$$

(9c) $$z_{min}\leq z^{i}\left(\left.k\right|t\right)\leq z_{max}$$

(9d) $$u_{min}\leq u^{i}\left(\left.k\right|t\right)\leq u_{max}$$

(9e) $${\Delta u}_{min}\leq u^{i}\left(\left.k\right|t\right)-u^{i}\left(\left.k-1\right|t\right)\leq {\Delta u}_{max}\;\left(-b_{i}{\left(z^{i}\left(\left.k\right|t\right)\right)}^{T}\lambda_{ij}\left(\left.k\right|t\right)-b_{j}{\left(z^{j}\left(\left.k\right|t\right)\right)}^{T}\mu_{ij}\left(\left.k\right|t\right)\right)\geq d_{min},$$

(9f) $$A_{i}{\left(z^{i}\left(\left.k\right|t\right)\right)}^{T}\lambda_{ij}\left(\left.k\right|t\right)+s_{ij}\left(\left.k\right\rfloor t\right)=0,\;A_{j}{\left(z^{j}\left(\left.k\right|t\right)\right)}^{T}\mu_{ij}\left(\left.k\right|t\right)-s_{ij}\left(\left.k\right\rfloor t\right)=0,\;\left\|s^{ij}\left(\left.k\right|t\right)\right\|\leq 1,\;\;\;\;\;-\lambda_{ij}\left(\left.k\right|t\right)\leq 0,\;\;\;\;\;-\mu_{ij}\left(\left.k\right|t\right)\leq 0,\;\;\;\;\;for\;all\;i\in V,j\in N_{i}$$

where V is the number of the vehicles, and $N_{V}$ refers to the size of the platoon. Equation (9a) is the state variable which is a discretized form of the vehicle kinematic bicycle model that we are using in the MPC (Equations (5)–(8)), and $z_{min}$ and $z_{max}$ represent the boundaries for these states. $u^{i}\left(\left.k\right|t\right)$ is the control input which is a combination of acceleration/braking and steering of the i-th vehicle at k-th step predicted at time t and $u_{min}$ and $u_{max}$ are the limits on the inputs. $A_{i}$ and $b_{i}$ are the polytopic set of the i-th vehicle at step k predicted at time t. Also, $A_{j}$ and $b_{j}$ represent the polytopic set of the j-th vehicle which belongs to the surrounding vehicles set $N_{i}$. This polytopic representation is time-varying and is a function of the vehicle state at each time step (see Appendix A). $\Delta u^{i}\left(\left.k\right|t\right)$ is the jerk minimization (change in control input in two consecutive time steps) for comfort and energy saving. Therefore, Equation (9e) avoids heavy braking/acceleration as well as aggressive steering and enhances energy efficiency and comfort and Equation (9f) is to guarantee collision avoidance. The set of neighbors $N^{i}$ is the set of all the vehicles within the platoon except i-th vehicle and is defined as $N^{i}$. In order to guarantee collision avoidance, the vehicles are modeled as polytopic sets in which not only each set has an empty intersection with all the other sets, but also each set keeps a minimum distance from the other sets.

$Q_{z},\;Q_{u},\;Q_{\Delta u}$ are positive-definite weight matrices that penalize, respectively, the tracking error, control effort, and rate of change in control input. The objective function aims to minimize the deviation of each vehicle from the reference trajectory (that is generated by the offline planner) and minimizing inputs and jerk. The acceleration and steering inputs are computed during the horizon. The first control input is applied to calculate the state variable. For the next time step, the initial state is updated and the procedure of solving the optimization problem of Equation (9) subjected to Equation (9a)–(9f) constraint is repeated over the platooning time.

Solving Equation (9) subject to the corresponding constraints renders the inputs to the vehicles at different times. The first input at time t is then implemented in the bicycle model and the state will be updated. The merging time is discretized with a time step of 0.1 s, meaning that vehicle states are updated every 0.1 s of over the time to form the platoon. This resolution strikes a balance between computational efficiency and sufficient temporal accuracy for tracking smooth trajectories.

Unless stated otherwise, all simulations use the parameters listed in Table 1.

### 2.3. Energy Saving

Regulations on vehicle energy efficiency have become stricter and thus, the research and application of vehicle energy-saving methods have become critical [42,43]. The energy-saving can be analyzed from aerodynamic and vehicle speed or acceleration optimization perspectives [31,32,44]. Energy-saving methods based on aerodynamics include field tests and wind-tunnel tests, and these are out of the scope of this paper. Vehicle speed optimization mainly focuses on optimization of the acceleration/braking behavior of vehicles in the platoon. Designing a control system that optimizes the acceleration/braking behavior of the vehicle can result in maximum efficiency working conditions, and subsequently maximum energy saving. In recent years, the MPC has become an important research direction of vehicle platoon control that can predict the vehicle control input [16,17].

To enable energy-aware analysis within the centralized MPC framework, the acceleration/braking of each vehicle is explicitly computed as part of the control input and optimized at each time step within the prediction horizon. Since energy consumption is closely related to the magnitude and the variability of acceleration/braking, minimizing this input within the optimization problem indirectly promotes energy efficiency. By extracting and analyzing the acceleration/braking profiles across different scenarios (prediction horizons), we can quantitatively assess the control strategy’s impact on potential energy usage. To obtain the energy consumption per mass (i.e., energy density) of the vehicle, the driving force per mass (i.e., acceleration) is simply integrated over the distance and course of the time to form the platoon [45,46]:

(10) $$Energy\;density=\int \left|a(t)\right|\cdot dx\cdot dt$$

## 3. Results and Discussion

The nonlinear optimization problem described in Equation (9) along with the model described in Equations (5)–(8) were formulated in PYOMO and solved using IPOPT for platoons of different sizes (two, four, and six vehicles) to evaluate the effectiveness of the proposed merging algorithm.

Initially, the vehicles are moving into two different adjacent lanes and a platoon is formed after all vehicles merge into the bottom lane. For example, for a platoon with four vehicles (Figure 2), the vehicles are initially positioned at the longitudinal coordinate of [*x*^1^(0), *x*^2^(0), *x*^3^(0), *x*^4^(0)] = [0, 8, 0, 8] and the lateral coordinates of [*y*^1^(0), *y*^2^(0), *y*^3^(0), *y*^4^(0)] = [0, 0, w, *w*], where w = 3.7 m is the width of the lane (the distance between the middle of the upper and bottom lanes), accommodating realistic driving conditions. After forming the platoon, *y* = 0 for all the vehicles. In the offline controller, we generated the reference trajectory and this trajectory is fed into the online controller. At the first time step, the controller uses the initial state of all vehicles to solve the optimization problem (Equation (9)) along with the model dynamics (Equations (5)–(8)). This provides a sequence of optimal control inputs, from which only the first input is applied. The vehicle states are then updated online, and the optimization problem is resolved at the next time step in a receding-horizon manner. The dimensions of the vehicles were set to 4.5 m in length and 1.8 m in width, which are typical of a mid-size vehicle. Control input limits were set to realistic values to ensure practical applicability: the maximum acceleration was limited to ±0.5×g m/s^2^, with the rate of change between acceleration inputs restricted to ±0.2×g m/s^2^ per time step; the steering angle was limited to ±45°, and its rate of change was limited to 10 °/s. $d_{min}$ is chosen as 1 m. The reference velocity of 17 m/s is considered in all the simulations unless otherwise specified.

Depending on the size of the platoon and other factors, it takes approximately between 2.5 to 4 s for each lane changing to be completed. During this time, the vehicles adjust their positions and speeds to align into one lane, ensuring no collisions. Using trial and errors, we found out that for a system with two vehicles, the prediction horizon that makes the solution feasible should be at least 18, which corresponds to predicting 1.8 s in advance. For prediction horizons below this minimum value, the controller deals with a shortsighted situation, and a safe platooning is unfeasible. This means that the controller cannot find any sequence of control inputs over the horizon range [0, *N*] that leads to a feasible path while satisfying all constraints. This minimum horizon was found to be 20 and 30 for platoons with four and six vehicles, respectively, predicting 2.0 and 3.0 s in advance.

### 3.1. Coordinated Maneuvering and Dynamics Across Platoon Sizes

#### 3.1.1. Results for Platoon Size 2

**Figure 4:**
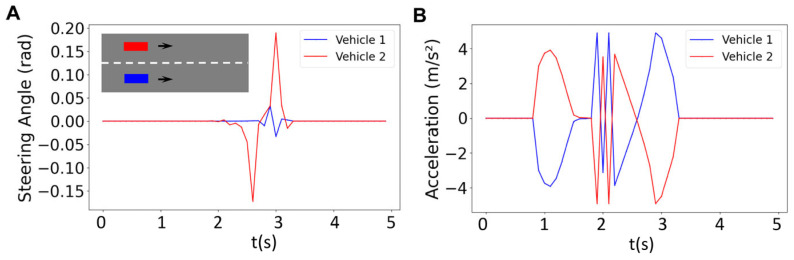
Results for the steering angle (**A**) and acceleration (**B**) of two vehicles for the horizon of *N* = 18.

**Figure 5:**
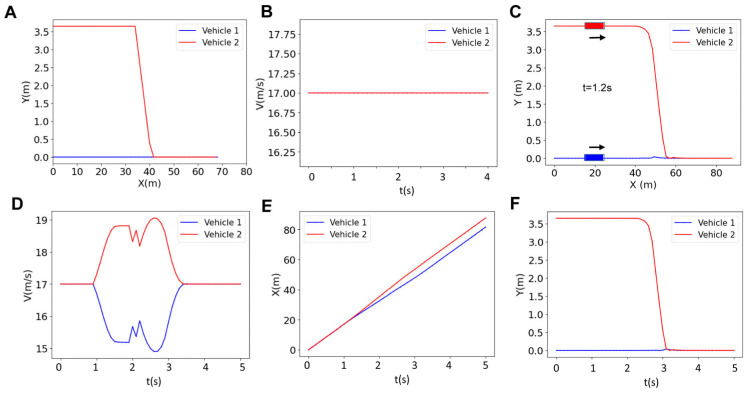
Results for the reference trajectory (**A**), reference velocity (**B**), online trajectory (**C**), online velocity (**D**), longitudinal position (**E**), and lateral position l (**F**) of two-vehicle platoon for the horizon of *N* = 18.

In order to control the vehicles, we need to calculate the inputs using the optimization problem described above (Equation (9)). These inputs include the steering angle and acceleration/braking. The inputs for a platoon with two vehicles are depicted as a function of time in Figure 4. These inputs are used to generate the corresponding states (i.e., longitudinal position, lateral position, heading angle, and velocity) that are depicted in Figure 5. The graph for the blue vehicle shows approximately a uniform steering angle, indicating that it is maintaining a straight path (staying in its lane). However, the red vehicle undergoes changes in direction twice which correspond to the two turns that the vehicle makes to merge into the bottom lane (Figure 4A). The first turn represents a lateral shift from the upper lane toward the bottom lane and the second turn happens when the vehicle realigns its heading to follow the straight path along the bottom lane. The blue vehicle shows a zero-acceleration profile until about t = 0.8 s, when it starts to brake to make space for the red vehicle merging into the bottom lane (Figure 4B). The trend of acceleration magnitude for the red vehicle is almost identical but the directions are opposite, meaning that when the blue vehicle decelerates to create space, the red vehicle accelerates to occupy the space created by the blue vehicle for merging. The first blue peak at t = 1 s in Figure 4B represents braking by the blue vehicle to reduce velocity and create space. This is simultaneous with the red vehicle accelerating (first red peak at t = 1 s in Figure 4B) to occupy the created space (the blue vehicle slows down while the red vehicle accelerates). After this period, the vehicles achieve their maximum magnitude at around t = 1.5 s (also see Figure 5A–D). Vehicles then execute two fast accelerating and braking actions between t = 1.8 s and t = 2.2 s. These occur prior to the critical phase of the merging maneuver (see Figure 5F). This is a result of the controller reacting to strict collision avoidance and spacing constraints within a finite prediction horizon before merging. Then at t = 2.2 s (right before merging starts), the blue vehicle accelerates and the red vehicle decelerates to start a safe merging (Figure 5F). The vehicles finally recover the reference velocity (t = 2.8–3.25 s) by decelerating (blue) and accelerating (red) to form a stable platoon and there is no significant acceleration or braking afterward (t > 3.25 s) (Figure 4B and Figure 5D,F).

Figure 5 illustrates the results for the reference trajectory and velocity, the online trajectory and velocity, as well as longitudinal and lateral positions of vehicles in a platoon of size 2 with a prediction horizon of *N* = 18 (i.e., all vehicles are controlled using an MPC with a prediction horizon of *N* = 18). We observed that the reference trajectory (Figure 5A) is different from the online trajectory (Figure 5C), although the overall trend of online trajectory follows the reference trajectory. This is due to the fact that the prediction horizon as well as the collision avoidance constraints are updating the reference trajectory to form the online trajectory. Initially, both vehicles travel at similar velocities (zero acceleration as in Figure 4B) and keep close longitudinal positions, indicating coordinated movement. However, at t~0.9 s, the blue vehicle decreases its velocity while the red vehicle speeds up (Figure 4B and Figure 5D). The overall platoon formation is complete in approximately 3.5 s, demonstrating the effectiveness of the centralized control system in managing vehicle coordination and safety within the given prediction horizon.

#### 3.1.2. Results for Platoon Size 4

**Figure 6:**
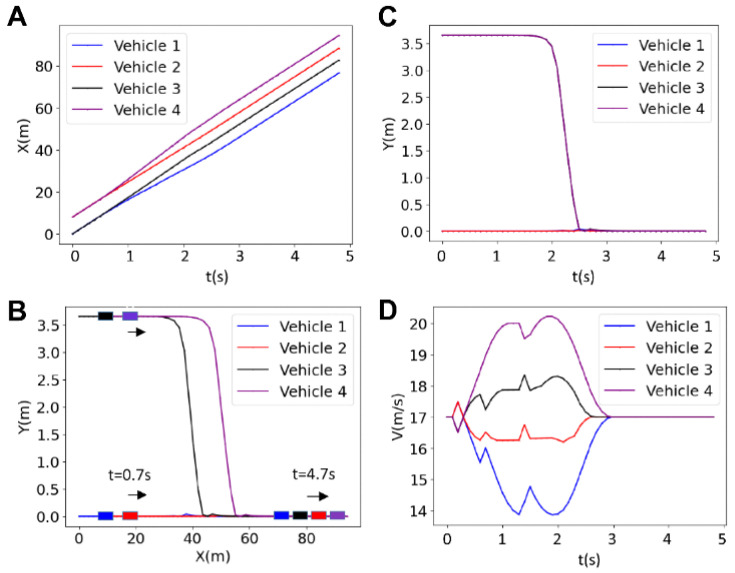
Results for the longitudinal position (**A**), lateral position (**B**), trajectory (**C**) and the velocity (**D**) of four vehicles for the horizon of *N* = 20.

Figure 6 presents the results for the longitudinal and lateral positions, as well as the trajectory and velocity in a platoon of four vehicles with a prediction horizon *N* = 20. The figure illustrates how the vehicles adjust their velocities and positions to maintain a safe platoon formation. Initially, the vehicles travel at the same velocity, but in a synchronous manner; they gradually adjust their velocities by accelerating (purple and black vehicles), or decelerating (red and blue vehicles) to create necessary space between them before forming the platoon (Figure 6D). This leads to a gradual increase in the distance between the vehicles, which starts at t~0.3 s and becomes a visible diverging bifurcation between x-t graphs at ~0.7 s as shown in Figure 6A. It should be noted that fluctuations occurring in the velocities at t = 1.4 s arise from quick braking (purple) and accelerating (red, black, and blue) to adjust the inter-vehicle distance online. The entire platoon formation is complete in approximately 3 s.

#### 3.1.3. Results for Platoon Size 6

**Figure 7:**
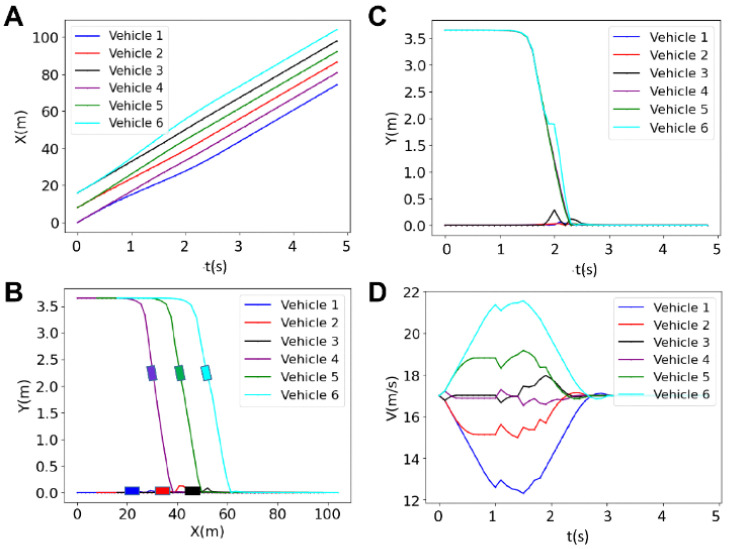
Results for the longitudinal position (**A**), lateral position (**B**), trajectory (**C**) and the velocity (**D**) of 6 vehicles for the horizon of *N* = 30.

Figure 7 depicts the results for the longitudinal and lateral positions, as well as the velocity, of six vehicles in a platoon with a prediction horizon of *N* = 30. The figure shows how the vehicles adjust their trajectories and speeds to form and maintain a coordinated platoon over time. Initially, there are variations in the velocities of the vehicles, but as time progresses, the vehicles synchronize their movements to aid the platoon formation. The cyan vehicle increases its velocity while the black and purple vehicles keep their velocity uniform, and other vehicles make necessary adjustments to their speeds. While the blue and red vehicles decrease their velocities, the cyan and green vehicles increase their velocities to provide sufficient gaps between each vehicle, ensuring safe distances are maintained during the lane merging operation. These adjustments are crucial for preventing collisions and ensuring a smooth and efficient formation of the platoon. The figure emphasizes the importance of dynamic velocity adjustments and careful coordination among the vehicles. The platoon formation is completed in less than 2.4 s, demonstrating the effectiveness of the centralized control system in managing multiple vehicles.

### 3.2. Effect of Prediction Horizon on Merging Sequence

**Figure 8:**
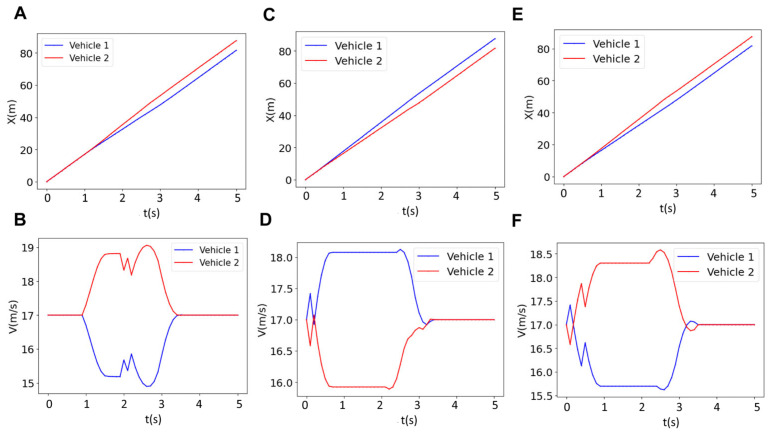
Results for the longitudinal position (left column) and the velocity (right column) of two vehicles for three different horizons of (**A**,**B**) *N* = 18, (**C**,**D**) *N* = 30, and (**E**,**F**) *N* = 40.

The results of the longitudinal position and velocities of a two-vehicle platoon for various prediction horizons (*N* = 18, 30 and 40) are depicted in Figure 8. The longitudinal value for the red vehicle for *N* = 18 (Figure 8A) and *N* = 40 (Figure 8E), which is merging from the upper lane to the bottom lane, is greater than that of the blue vehicle (x_blue_ < x_red_). This means that the red vehicle precedes the blue vehicle, which has been driving straight after forming the platoon. However, for *N* = 30, the blue vehicle precedes the red vehicle (x_blue_ > x_red_). This implies that the sequence of the vehicles depends on the prediction horizon. The vehicle order switching is rooted in the interplay between feasibility, optimality, and inter-agent constraints over a finite horizon. Specifically, the MPC solves a multi-agent optimization problem where each vehicle’s state and input trajectories are coupled through shared constraints, and the dual variables (Lagrange multipliers) reflect the system’s interaction structure. When the prediction horizon changes, the solution space and the balance between cost minimization and constraint satisfaction are altered. This affects both the optimal trajectories and the associated dual variables, leading to a different resolution of priority between vehicles. In essence, the reordering emerges from the horizon-dependent structure of the optimization problem itself. This reordering in vehicles, which is captured by the present online algorithm, could not be captured by offline methods. This is significant for controller design, especially in scenarios where a specific vehicle is designated to lead due to sensor range, Vehicle-to-Everything (V2X) communication capabilities, or driving authority (e.g., human-supervised vs. autonomous). In such cases, tuning the prediction horizon can directly influence the resulting vehicle sequence. For this change in the sequence of vehicles, the sign of relative velocity of the vehicles should be altered (V_blue_ − V_red_ > 0) so that one can precede the other. It should be noted that at *N* = 18, the controller is “shortsighted.” It optimizes for immediate performance, potentially allowing the red vehicle to merge aggressively and temporarily overtake the blue vehicle because the long-term consequences (e.g., platoon stability) are not fully considered. At *N* = 40, the controller is overly cautious. It may prioritize strict adherence to the reference velocity or platoon formation over longer timescales, leading to conservative merging behavior. At *N* = 30 maintains a balance. The controller can foresee enough steps to coordinate merging and velocity matching optimally, ensuring the blue vehicle naturally precedes the red vehicle without aggressive or overly conservative maneuvers.

To analyze the velocity variations, one can refer to Figure 8B,D,F where the velocities of the two vehicles are plotted as a function of time. As can be seen, for *N* = 18 and *N* = 40, the red vehicle increases its velocity to around 19 m/s, while the blue vehicle decreases its velocity to around 15.5 m/s. This scenario involves more frequent acceleration and braking. However, for *N* = 30, the velocities are closer to the reference velocity (i.e., 17 m/s) compared to the *N* = 18, *N* = 40; the blue vehicle’s velocity increases to around 18 m/s, and the red vehicle’s velocity decreases to around 16 m/s. The less frequent acceleration and braking associated with the optimal horizon *N* = 30 implies that this case saves energy, which will be discussed in detail in the next sections. Additionally, the velocity profile for *N* = 30 is flatter than *N* = 18 and *N* = 40, suggesting that there is minimal acceleration and braking at *N* = 30. These results overall suggest that *N* = 30 is optimal for tracking the reference velocity or achieving efficient braking and acceleration. The optimal horizon for energy saving will also be discussed in detail in the next section.

To further quantify the impact of the prediction horizon on the platoon sequence, Table 1 summarizes the resulting vehicle order for a four-vehicle platoon at horizons *N* = {20,25,30,35}. Table 2 shows how the platoon order changes with the horizon length. The sequence indicates that horizons allow vehicles to reconfigure during merging. Such reordering reduces overall cost and has implications for authority: depending on deployment, the operator may enforce fixed-leader, lane-aware, or utility-driven policies.

### 3.3. Optimal Prediction Horizon for Stable and Energy Efficient Platooning

**Figure 9:**
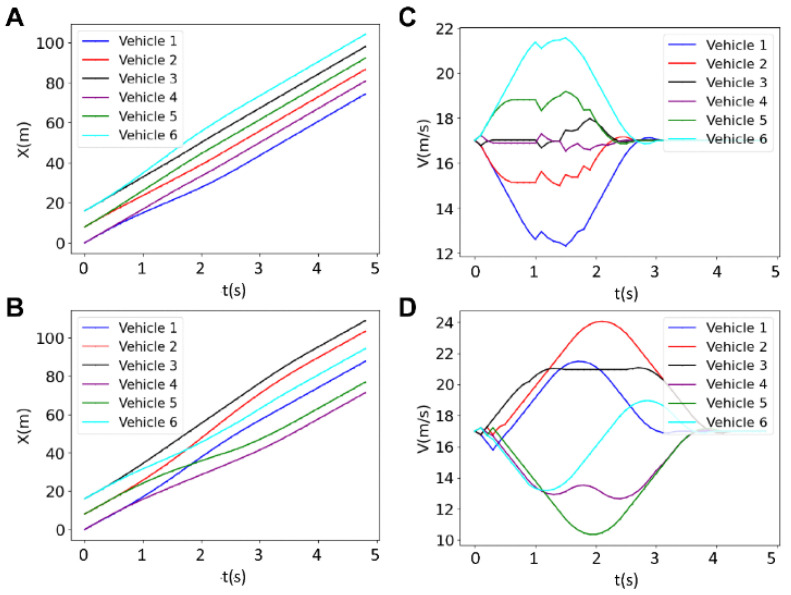
Results for the longitudinal position and the velocity of six vehicles in the platoon for two different horizons of *N* = 30 (**A**,**C**) and 40 (**B**,**D**).

The effect of the prediction horizon on the longitudinal position and the velocity of the vehicles for a six-vehicle platoon is shown in Figure 9. The velocity profiles for *N* = 30 show that four vehicles adjust their speeds relatively fast (t = 0.1–0.5 s for red and green, and t = 0.1–0.9 s for blue and cyan) to reach a steady velocity. The black and purple vehicles keep a nearly steady velocity close to the reference velocity throughout the course. For *N* = 40, however, the velocity plot exhibits more pronounced fluctuations and longer time of velocity adjustment for all vehicles (t = 0–4 s). This increase in the time course of velocity adjustment is due to the fact that at a higher horizon, the decision variables need to be computed over more time steps and this makes the controller slower to reach the steady condition. Additionally, the fluctuations in the velocity are a result of the change in the sequence of vehicles in the platoon, which was discussed for a two-vehicle platoon in Figure 8. In a real-world application, at a higher horizon, the vehicles experience greater changes in speed before settling, indicating the excessive accelerating and braking. This could result in higher energy consumption and less smooth driving experiences. This finding emphasizes the importance of selecting an appropriate prediction horizon to optimize the performance and efficiency of an online centralized MPC in platooning.

**Figure 10:**
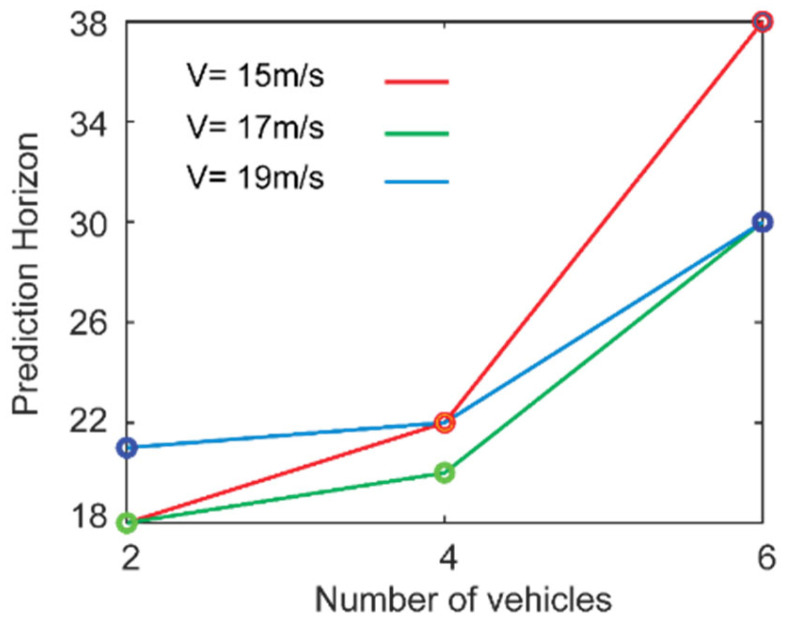
Prediction horizon as a function of number of vehicles and at various reference velocities V = 15, 17, and 19 m/s.

The minimum prediction horizon that ensures feasibility of platooning is plotted as a function of the size of the platoon and reference velocity in Figure 10. We observed that regardless of the reference velocity, as the size of the platoon increases, the prediction horizon increases, implying that for a higher number of vehicles a longer prediction time is needed by the vehicles to merge into one lane. Additionally, we also observed that for a lower reference velocity (15 m/s), there is a sharper rise in the horizon as the number of vehicles in the platoon increases. However, at higher reference velocities the horizon becomes less dependent on the number of vehicles. For the reference velocity of 15 m/s, the system requires a relatively low minimum prediction horizon when only two vehicles are involved. However, as the number of vehicles increases, the required horizon rises significantly—reaching the highest value among all tested cases. This is because lower velocities lead to vehicle accumulation and denser traffic during the merging process. With more vehicles, this increases the likelihood of congestion and potential collisions, requiring a longer prediction horizon to safely coordinate maneuvers and maintain feasibility. It should be noted that below a certain value prediction horizon, the optimizer does not deliver any solution such that constraints are not violated. This is because the system becomes shortsighted and no collision-free situation exists. This has been tested in our simulation by assigning different values of prediction horizon and checking the feasibility of the solution, showing that for two-, four-, and six-vehicle platoons, the minimum horizons for feasibility are 18, 20, and 30, respectively.

**Figure 11:**
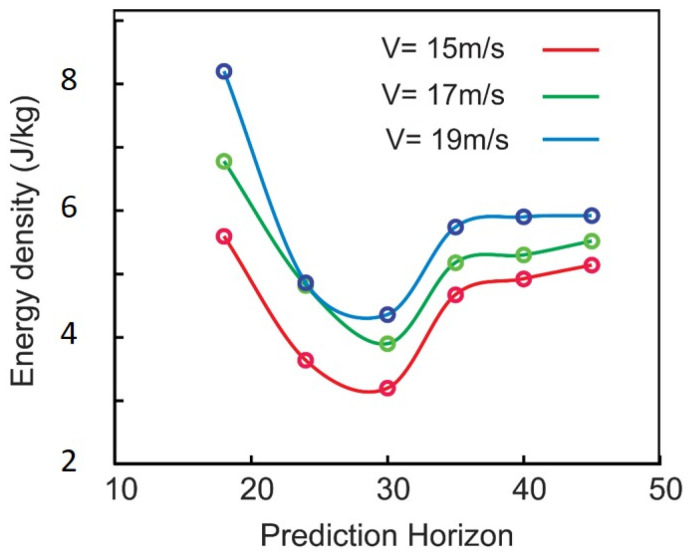
Energy consumed per mass of the vehicle for two-vehicle platoon and at different reference velocities and prediction horizon.

It was then hypothesized that the prediction horizon might have an interesting effect on the energy consumed by the vehicles in the platoon. To obtain the kinematic energy consumption per mass of the vehicle during the braking/acceleration phase, the driving force per mass (i.e., absolute acceleration) is integrated over the distance and time course of the simulation ($\int \left|a\left(t\right)\right|\cdot dx$) [45]. Figure 11 illustrates how choosing the prediction horizon affects the energy consumption (energy per mass of the vehicle) in a platoon with two vehicles. Surprisingly, there is an optimal prediction horizon that minimizes the energy consumption of the vehicles. As shown in the figure, selecting an appropriate prediction horizon can result in energy savings of 35–40%. This optimal value exists regardless of the speed, and it does not significantly change with the speed. This is very important from the energy efficiency standpoint, as it means that tuning the prediction horizon can optimize the fuel consumption and save energy. While prior studies have demonstrated meaningful energy reductions using a fixed-horizon MPC or offline trajectory planning—such as 12% savings in [33], 16.1% in [35], and 21.3% in [36]—our proposed method achieves up to 35–40% energy savings through prediction horizon tuning. Further energy reduction beyond our results would require more hardware-specific optimization, which falls outside the scope of the current MPC-based coordination.

While this study assumes a fully autonomous vehicle environment, the proposed centralized MPC framework can be extended to mixed traffic scenarios with both autonomous and human-driven vehicles. In a mixed environment, human drivers exhibit higher uncertainty and less predictable behavior compared to fully automated agents. Shorter horizons allow for more immediate, aggressive merging maneuvers, suitable for interactions with impatient drivers. Longer horizons enable more cautious, conservative merging behavior, increasing safety margins when interacting with unpredictable or defensive drivers. Additionally, medium-length horizons often achieve the best energy efficiency by balancing early planning with smooth merging trajectories. However, the variability in the prediction horizon inherent in the proposed method provides a pathway for adaptation: for example, an autonomous vehicle could select a longer prediction horizon to allow safer, more conservative planning when interacting with unpredictable human-driven vehicles. Moreover, collision avoidance constraints can be made more conservative by dynamically adjusting safe margins based on estimated human behavior.

### 3.4. Robustness to Disturbance

**Figure 12:**
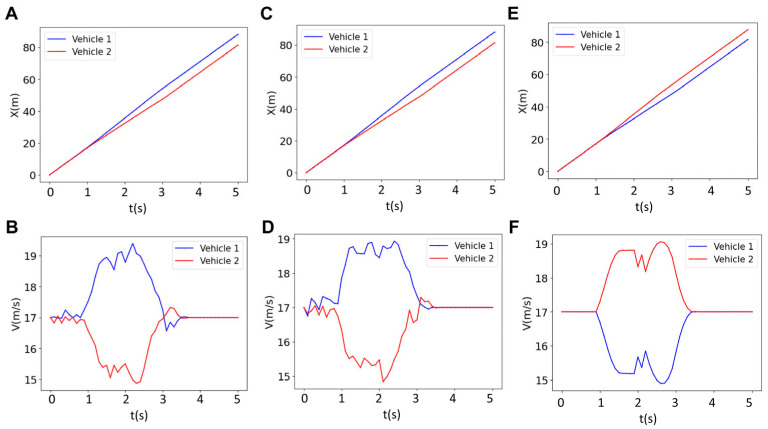
Results for the trajectory and the velocity of two vehicles in the platoon for model uncertainty (**A**,**B**), actuator/sensor noise (**C**,**D**) and no noise/no uncertainty (**E**,**F**) cases at *N* = 18.

To evaluate the robustness of the proposed centralized MPC framework under realistic conditions, we introduced sensor and actuator noise and unmodeled dynamics. Under such disturbances, the optimization problem (Equation (9) does not render any feasible solution for 1m minimum inter-vehicle distance (*d_min_*) that was used for the no-disturbance case. To obtain a feasible solution, we adjusted the minimum inter-vehicle distance by increasing it from 1.0 m (used in the no-disturbance cases) until we achieved a feasible solution at 1.6 m. This revealed that the minimum safety distance should not be treated as a fixed parameter, but rather as a design variable that must be adapted based on the level of disturbance. The results for the trajectory and velocity of the vehicles with and without disturbance are depicted in Figure 12. We observed that the velocity of the vehicle has fluctuations, but merging occurs without any collision.

To further analyze this effect, we parametrized the minimum feasible inter-vehicle distance *d_min_* with respect to different noise levels. Table 3 reports the values of *d_min_* required to maintain feasibility under low, medium, and high noise. The results show a nonlinear increase in the required safety margin as noise grows, highlighting the inherent trade-off between robustness and roadway efficiency.

**Figure 13:**
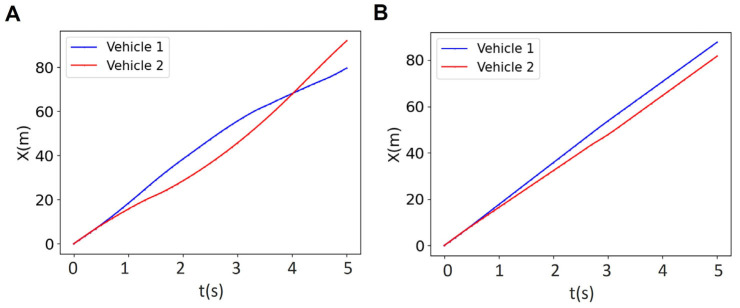
Sensitivity of the MPC to reference trajectory. (**A**) Larger longitudinal offsets remained feasible but required longer horizons (*N* = 24). (**B**) A perturbed sub-optimal reference was corrected successfully, with only a modest increase in computational effort.

To evaluate the effect of the reference trajectory quality on controller performance, we tested both larger longitudinal offsets and perturbed sub-optimal references. As shown in Figure 13, trajectories with larger offsets remained feasible but required longer prediction horizons (e.g., *N* = 24 vs. *N* = 18). In addition, the MPC successfully corrected a perturbed sub-optimal reference, preserving feasibility with only a modest increase in computational effort. These results confirm that the proposed framework is robust to non-ideal reference trajectories, though more complex references may increase computational demand.

### 3.5. Computational Complexity and Scalability

The computational burden of the centralized MPC grows with both platoon size and prediction horizon. To assess scalability, we measured average IPOPT solver times for different platoon sizes (two, four, and six vehicles) and prediction horizons (18, 30, and 40). The results are reported in Table 4. The iteration time increases nonlinearly with the number of vehicles, reflecting the polynomial growth in decision variables and constraints. Nevertheless, the solution remained feasible for all tested cases, satisfying real-time requirements for up to six vehicles. Based on these trends, we anticipate a significantly higher computational effort for larger platoons (e.g., >10 vehicles), where the “real-time” claim may not strictly apply. In such scenarios, the controller should be more appropriately described as an online solver, and distributed MPC formulations may be required for scalability. This analysis positions the centralized MPC as a benchmark feasibility study for small platoon sizes, providing insight into its practical limits.

### 3.6. Effect of Road Friction

Since the kinematic bicycle model does not capture slip dynamics or detailed tire–road interactions, we evaluated the framework’s performance under reduced friction to approximate wet-road conditions. In this validation, the friction coefficient was set to μ = 0.3, with acceleration constraints adjusted to represent low-friction surfaces.

**Figure 14:**
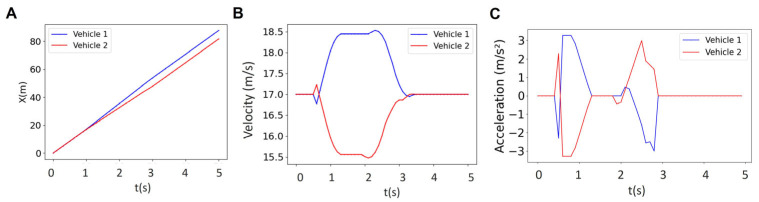
Results for the longitudinal position (left column), velocity (middle column), and acceleration (right column) of two vehicles for low friction (**A**–**C**) at *N* = 23.

Figure 14 presents the longitudinal position, velocity, and acceleration of two vehicles under these conditions with a prediction horizon of *N* = 23. The results show that the centralized MPC framework remained feasible and stable, and the controller maintains effectiveness under low friction.

Nevertheless, for high-speed driving or aggressive maneuvers, where tire forces and slip become dominant, a dynamic vehicle model would be required to accurately capture road–tire interactions. The present kinematic formulation remains appropriate for the urban-speed platooning scenarios considered in this work.

### 3.7. Communication Delay Effects

In practical implementations, vehicle-to-vehicle (V2V) and vehicle-to-everything (V2X) communication are subject to delays, packet loss, and asynchrony. To evaluate the impact of imperfect communication, we introduced a 0.1 s communication delay into the centralized MPC framework.

**Figure 15:**
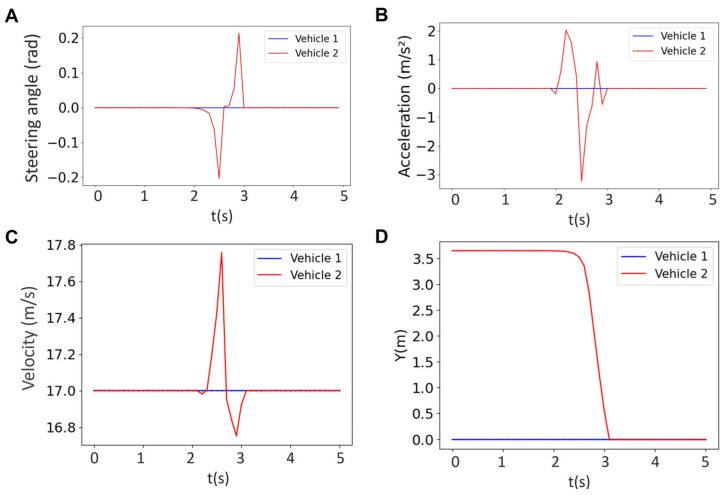
Results for the steering angle (**A**), acceleration (**B**), velocity (**C**), lateral position (**D**) of a two-vehicle platoon for the horizon of *N* = 24 under the communication delay of 0.1 s.

Here, we show in Figure 15 that our MPC can address the communication delay. Figure 15 illustrates the results for the steering angle, acceleration, velocity, and lateral position of a two-vehicle platoon under this delay at a prediction horizon of *N* = 24. We observed that the non-maneuvering vehicle (Vehicle 1, blue) maintained nearly constant velocity and control efforts.

Importantly, the minimum feasible prediction horizon increased from *N* = 18 (no delay) to *N* = 24 when the delay was included. This reflects the need for a longer look-ahead in the presence of delayed information to ensure constraint satisfaction and maintain safety. These results suggest that a centralized MPC can remain feasible under moderate delays.

## 4. Sensitivity Analysis

The performance of the proposed centralized MPC method is influenced by several key parameters including the prediction horizon (N), computational sampling time (dt), reference velocity, initial vehicle spacing, and the number of vehicles. The prediction horizon directly affects both feasibility and energy optimization: too short a horizon leads to unfeasibility or unsafe merging, while excessively long horizons increase the computation time without energy efficiency or safety advantages. The sampling time (dt) must balance control resolution and computational efficiency. A smaller dt allows finer control and better tracking accuracy, but increases the number of decision variables and slows down computation. Conversely, a larger dt reduces the size of the optimization problem, but may degrade control precision and may result in more abrupt control inputs. We performed trial and error to obtain the optimal computational time step (dt) and found that values greater than 0.1 s led to unsmooth results and that values smaller than 0.1 increased the computational time without improving the accuracy of the results. Therefore, 0.1 s was chosen as the time step for the simulations. Reference velocity also plays a crucial role, with higher velocities requiring longer prediction horizons to maintain feasibility and safe merging (see Figure 10). Additionally, initial vehicle spacing affects the ease of coordination, with tighter initial spacing making collision avoidance constraints harder to satisfy. Overall, prediction horizon tuning, velocity setting, and inter-vehicle spacing are the most critical factors influencing the controller’s effectiveness.

Increasing the prediction horizon *N* in our MPC formulation can be interpreted as a form of input/command shaping, where acceleration and braking commands are effectively filtered to reduce oscillations. This behavior is closely related to string stability in vehicle platoons, which can be modeled as mass–spring chains that are prone to disturbance amplification. In this analogy, larger horizons allow the controller to act as a prediction-based filter, damping oscillations and thereby improving string stability across the platoon.

## 5. Limitation and Future Work

While the proposed online centralized MPC framework demonstrates strong performance for urban lane merging, several limitations should be acknowledged to guide future extensions:

### 5.1. Vehicle Modeling Assumptions

This study adopts a kinematic bicycle model, which is well established and sufficiently accurate for urban-speed scenarios where slip angles and tire forces remain limited. At higher velocities or under aggressive maneuvers (e.g., sharp braking or operation on icy surfaces), dynamic vehicle models would be required to capture road–tire interactions.

### 5.2. Communication Constraints

Our centralized approach assumes reliable vehicle-to-vehicle communication. Preliminary tests with communication delays show feasibility with modest adjustments to prediction horizons, but in large-scale or latency-prone networks, packet loss and asynchrony may significantly affect performance. Incorporating realistic communication models and developing delay-tolerant MPC formulations would improve robustness for deployment in real traffic networks.

### 5.3. Scalability to Large Platoons

The framework remains computationally tractable for platoons of up to six vehicles and can achieve online operation in such cases. However, as the number of vehicles grows, the computational burden increases nonlinearly. While a centralized MPC serves as a valuable benchmark for safety-critical merging, scaling to larger fleets (>10 vehicles) will likely require distributed formulations that balance tractability with coordination.

### 5.4. Reference Trajectory Quality

The method depends on an offline-generated reference trajectory, which is corrected online by MPC. Sensitivity analysis shows robustness even to perturbed or suboptimal references, though longer prediction horizons are sometimes required. Future work could integrate adaptive reference generation directly into the optimization loop, further reducing reliance on offline planning.

### 5.5. Robustness to Disturbances

Feasibility under disturbances was maintained by tightening the minimum safety distance. While this is a standard MPC practice, more systematic approaches such as a tube-based robust MPC or automated constraint tightening would provide stronger guarantees without manual tuning. Exploring such methods would strengthen robustness under uncertainty.

## 6. Conclusions

In the context of autonomous vehicles, proper lane merging is critical as it can reduce the traffic bottleneck and lead to safer road transportation. To obtain a collision-free and efficient lane merging, advanced control algorithms such as MPC need to be designed to smoothly coordinate multiple vehicles to form a platoon. Prior MPC-based lane merging studies mostly utilized offline planning with a precomputed lookup table of feasible maneuvers to model lane merging. Although these model designs reduce the online computational load, they lack flexibility, as they rely on predefined scenarios and cannot easily adapt to dynamic or unpredictable situations. In this study, we proposed an online centralized MPC framework for robust lane merging for multiple autonomous vehicle scenarios. To evaluate the flexibility of our online algorithm, we examined the role of the prediction horizon, platoon size, and disturbances in determining both the feasibility and efficiency of merging maneuvers. To achieve this, the nonlinear optimization problem, comprising the dynamic model and constraints, was written in a discrete-time format using the forward Euler method within PYOMO, and solved using IPOPT solver. Our results revealed that there exists an optimal prediction horizon at which braking and acceleration can be minimized, thereby reducing energy consumption by 35–40%. Additionally, we observed that increasing the prediction horizon beyond the minimum required for feasibility can alter the vehicle sequence in the platoon. We also found that as the number of vehicles increased, the minimum feasible prediction horizon increased significantly.

## Figures and Tables

**Table 1:** Vehicle dimensions and control limits used in this study.

Parameter	Vehicle Length	Vehicle Width	Road Width	*d_min_*	Max/Min Acceleration	Max/Min Steering Angle	Max/Min Steering Rate Angle
Value	4.5 m	1.8 m	3.7 m	1 m	±0.5 g m/s^2^	±45°	±10°

**Table 2:** Order of the vehicles in a four-vehicle platoon versus the prediction horizon.

Horizon	4th	3rd	2nd	1st
*N* = 20	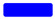	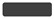	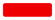	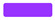
*N* = 25	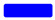	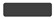	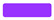	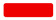
*N* = 30	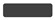	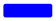	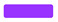	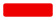
*N* = 35	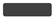	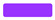	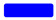	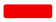

**Table 3:** Minimum feasible *d_min_* under different noise levels.

Noise Level	Low	Medium	High
*d_min_* (m)	1.3	1.6	2.3

**Table 4:** Iteration time (s) for various prediction horizons and platoon size.

Variable	Number of Vehicles (*N* = 30)			Horizon (2 Vehicle)		
Value	2	4	6	18	30	40
Iteration time (s)	0.03	0.053	0.09	0.016	0.03	0.057

**Figure A1:**
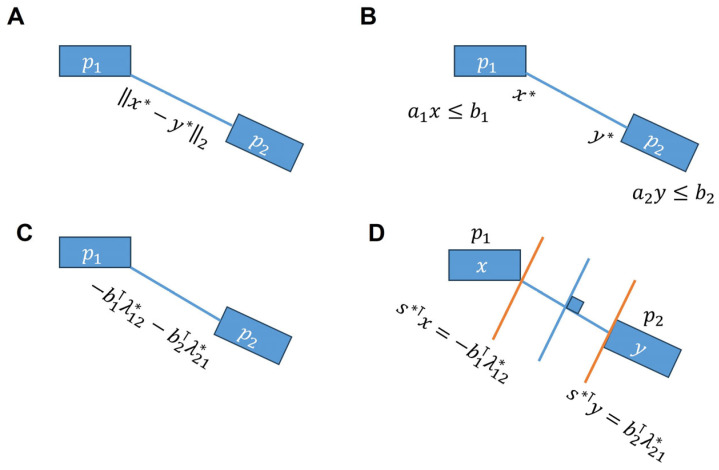
Illustration of collision avoidance using primal and dual formulations. Top (Primal): (**A**) computation of minimum distance, and (**B**) resulting optimal solution. Bottom (Dual): (**C**) minimum distance formulation, and (**D**) supporting hyperplanes ensuring non-intersection.

## Data Availability

The basic code supporting the findings of this study is available at Zenodo: http://doi.org/10.5281/zenodo.17074165.

## Abbreviations

The following abbreviations are used in this manuscript:

MPC	Model Predictive Control
CFTOC	Constraint Finite Time Optimal Control
IPOPT	Interior Point Optimizer (Solver)
V2V	Vehicle to Vehicle Communication
V2X	Vehicle to Everything Communication

## Appendix A

In multi-vehicle coordination, ensuring online collision avoidance is critical. One common method to achieve this is by formulating the collision avoidance constraint as a distance-based constraint between polytopes srepresenting each vehicle’s occupied space. This appendix provides a detailed mathematical formulation of how the vehicle trajectories are optimized under these constraints. Starting from the centralized MPC in Equation (A1), we reformulate the collision condition using dual variables. This method builds on prior works such as [14,47], and the key steps are presented in what follows. The basic formulation for the MPC optimization problem which we presented in Equation (9), can be written as:

(A1) $$\underset{u^{i}\left(\left.\cdot \right|t\right)}{\min}\sum_{i=1}^{N_{V}}\left(\sum_{k=t}^{t+N}{\left\|Q_{z}\left(z^{i}\left(\left.k\right|t\right)-z_{Ref}^{i}\left(\left.k\right|t\right)\right)\right\|}_{2}^{2}+\sum_{k=t}^{t+N-1}{\left\|Q_{u}\left(u^{i}\left(\left.k\right|t\right)\right)\right\|}_{2}^{2}+{\left\|Q_{\Delta u}\left(\Delta u^{i}\left(\left.k\right|t\right)\right)\right\|}_{2}^{2}\right)$$

Subject to

$$z^{i}\left(\left.k+1\right|t\right)=f\left(z^{i}\left(\left.k\right|t\right),u^{i}\left(\left.k\right|t\right)\right)\;z^{i}\left(\left.0\right|t\right)=z^{i}\left(t\right)\;z_{min}\leq z^{i}\left(\left.k\right|t\right)\leq z_{max}\;u_{min}\leq u^{i}\left(\left.k\right|t\right)\leq u_{max}\;{\Delta u}_{min}\leq u^{i}\left(\left.k\right|t\right)-u^{i}\left(\left.k-1\right|t\right)\leq {\Delta u}_{max}\;P\left(z^{i}\left(\left.k\right|t\right)\right)\cap P\left(z^{j}\left(\left.k\right|t\right)\right)=\emptyset ,i\neq j\;\mathrm{For}\;\mathrm{all}\;i\in V,j\in N^{i}$$

Here $P\left(z^{i}\left(\left.k\right|t\right)\right)$ is a collision avoidance constraint which represents i-th vehicle polytope as the road area occupied by the vehicle and $P\left(z^{j}\left(\left.k\right|t\right)\right)$ represents the other vehicle polytopes as moving obstacles for i-th vehicle. In our analysis, we replaced this constraint with those presented in Equation (9f).

The space occupied by the vehicle is defined by a two-dimensional convex polytope P which is called the pose of the vehicle here [47]. The pose of a vehicle is defined as $P\left(z\left(k\right)\right)=R\left(z\left(k\right)\right)P_{0}+t_{r}\left(z\left(k\right)\right)$ with $P_{0}$ being the initial pose and $z\left(k\right)$ the vehicle state at the k-th step in time. Here, R is an orthogonal rotation matrix, and $t_{r}$ is the translation vector. Since the vehicles can move in both lateral and longitudinal directions, the vehicle state z is two-dimensional. The rotation and translation of the vehicle respectively depend on the heading angle and position (x and y directions) of the vehicle. Therefore, the polytope can be rewritten as:

(A2) $$P\left(z\left(k\right)\right)=\left\{\left[p_{x},p_{y}\right]⊺\in \left.R^{2}\right|A\left(z\left(k\right)\right)\left[p_{x},p_{y}\right]⊺\leq b\left(z\left(k\right)\right)\right\}$$

where $p_{x}$ and $p_{y}$ are for the coordinates of points defining the polytope. The matrix $A\left(z\left(k\right)\right)$ and the vector $b\left(z\left(k\right)\right)$ are defined as [47]:

(A3) Azk=RψkT−RψkT,bzk=l2,ω2,l2,ω2T+Azkxk,ykT

where Rψk=cosψk−sinψksinψkcosψk. l and ω are the length and width of the vehicle, respectively. In the case of multiple vehicles, the distance between two consecutive vehicles is the difference between two polytopic sets representing those vehicles and can be written as:

(A4) $$dist\left(P_{1},P_{2}\right)=\underset{x,y}{min}\left\{\left.{\left\|x-y\right\|}_{2}\right|\;A_{1}x\leq b_{1},A_{2}y\leq b_{2}\right\}$$

where $P_{1}$ and $P_{2}$ are described by $A_{1}x\leq b_{1}$ and $A_{2}y\leq b_{2}$, respectively. In order to avoid collision, the distance between two consecutive vehicles should always be greater than zero. Here, we consider a minimum distance between each two consecutive vehicles (*d_min_*) as the constraint in the main optimization problem (Equation (9)) while this constraint itself serves as an optimization problem (Equation (A4)). Such a dual optimization problem can be formulated as follows [47]:

(A5) $$\underset{\lambda ,\mu ,s}{\max}\left\{-b_{1}^{T}\lambda -b_{2}^{T}\mu :A_{1}^{T}\lambda +s=0,A_{2}^{T}\mu -s=0,\;\left\|s\right\|\leq 1,\;\lambda ≽0,\;\mu ≽0\right\}$$

with λ, μ, and *s* denoting dual variables. The optimal value of the dual problem is the distance between the two polytopes $P_{1}$ and $P_{2}$ and is constrained to be larger than the minimum distance. In the polytopic framework, the edges adapt online to accommodate to changing positions and orientations of the vehicles, ensuring they always maintain safe separation. Dual variables act as weights that regulate the influence of each edge on avoiding inter-section occurrence between two vehicles. For example, when a vehicle is turning, the outer edges might require a stronger edge enforcement which is controlled by the dual variables. Incorporating the minimum distance constraint in the dual optimization problem yields the feasibility problem:

(A6) $$\exists \lambda ≽0,\mu ≽0,\;s:-b_{1}^{T}\lambda -b_{2}^{T}\mu \geq d_{min},A_{1}^{T}\lambda +s=0,A_{2}^{T}\mu -s=0,\left\|s\right\|\leq 1$$

The dual variables $\lambda_{ij}$, $\mu_{ij}$, and $s_{ij}$ are coupled through the collision avoidance constraint among vehicles i and j. $\lambda_{ij}\left(\cdot |t\right)$, $\mu_{ij}\left(\cdot |t\right)$, and $s_{ij}\left(\cdot |t\right)$ stand for the sequence of dual variables over the prediction horizon N. Therefore, the combined dual variables can be written as:

(A7) $$\lambda_{ij}\left(\left.\cdot \right|t\right)=\left\{\lambda_{ij}\left(\left.t\right|t\right),\ldots ,\lambda_{ij}\left(\left.t+N\right|t\right)\right\}\;\mu_{ij}\left(\left.\cdot \right|t\right)=\left\{\mu_{ij}\left(\left.t\right|t\right),\ldots ,\mu_{ij}\left(\left.t+N\right|t\right)\right\}\;s_{ij}\left(\left.\cdot \right|t\right)=\left\{s_{ij}\left(\left.t\right|t\right),\ldots ,s_{ij}\left(\left.t+N\right|t\right)\right\}$$

For clarity, the overall pipeline is illustrated schematically in Figure A1. This figure illustrates primal–dual coupling between two subsystems, p_1_ and p_2_. The primal variables x* and y* are connected through a shared consistency constraint, expressed as the Euclidean distance ${\left\|x^{*}-y^{*}\right\|}_{2}$. Each subsystem also satisfies its own local constraints, such as $a_{1}x\leq b_{1}$ and $a_{2}y\leq b_{2}$, which define their feasible regions in the primal problem. From these coupling constraints, dual variables $\lambda_{12}^{*}$ and $\lambda_{12}^{*}$ arise, entering the Lagrangian as terms like ${{-b}_{1}\lambda }_{12}^{*}{{-b}_{1}\lambda }_{21}^{*}$. These multipliers represent the prices of enforcing consistency between subsystems. At the optimum, the stationarity conditions of the dual problem ensure that the multipliers balance contributions from both subsystems, with equal and opposite forces maintaining the shared constraint between x and y. In this way, duality provides a bridge between local subsystem problems (p_1_, p_2_) and the global coordination problem: the primal side enforces feasibility of states, while the dual side enforces consistency through multipliers, ensuring alignment at the optimum.
